# Effects of NaHS and hydroxylamine on the expressions of brain-derived neurotrophic factor and its receptors in rats after cardiac arrest and cardiopulmonary resuscitation

**DOI:** 10.1186/s13049-018-0577-z

**Published:** 2018-12-22

**Authors:** Jiyan Lin, Weicheng Wu, Zhihong Xu, Siyao Liu, Wang Lu, Mandong Pan

**Affiliations:** Emergency Department, The First Affiliated Hospital of Xiamen University, No 55, Zhenhai Rd, Xiamen, 361003 China

**Keywords:** Hydrogen sulfide, Brain-derived neurotrophic factor, Tyrosine protein kinase B, p75 neurotrophin receptor

## Abstract

**Background:**

H_2_S can also protect nerve cells. The objective of the study is to investigate the effects of hydrogen sulfide (H_2_S) on the expressions of brain-derived neurotrophic factor (BDNF) and its receptors, tyrosine protein kinase B (TrkB) and p75 neurotrophin receptor (p75NTR), in brain tissues of rats with cardiac arrest and cardiopulmonary resuscitation (CA/CPR) following the restoration of spontaneous circulation (ROSC).

**Methods:**

Rats (*n* = 240) with CA/CPR were divided into three groups: Intervention (*n* = 80) that received sodium hydrosulfide (NaHS, 14 μmoL/kg·d) intervention after ROSC; Inhibition (*n* = 80) that received hydroxylamine (40 μmoL/kg·d) intervention after ROSC; and Control (*n* = 80) that received saline after ROSC. Kaplan-Meyer analysis was used to analyze the survival data. Quantitative real-time PCR (q-PCR), Western blot, immunohistochemistry and IODs (integrated optical density) were performed to determine the mRNA and protein expressions of BDNF, TrkB and p75NTR in rat brain tissues.

**Results:**

Survival rate of the three groups had significant difference (χ^2^ = 28.376, *p* = 0.000). The Intervention group had the highest survival rate (82.5%), while the Inhibition group had the lowest survival rate (62.5%). The mRNA and protein levels of BDNF and TrkB in the Intervention group were significantly higher compared to the Control group (*p* < 0.05); while the mRNA and protein levels of BDNF and TrkB in the Inhibition group was significantly lower than the Control group (*p* < 0.05) on days 1, 3, and 7. However, the mRNA and protein levels of p75NTR in the Intervention group were significantly lower than the Control group (*p* < 0.05); while the mRNA and protein levels of p75NTR in the Inhibition group were significantly higher than the Control group (*p* < 0.05) on days 1, 3, and 7.

**Conclusion:**

NaHS treatment increases the survival rate of rats after CA and ROSC by upregulating the expression and activation of BDNF and its receptor TrkB, and down-regulating p75NTR expression.

## Background

Post-cardiac arrest brain injury is a common cause of morbidity and mortality after cardiopulmonary resuscitation (CPR). Studies show that brain injury is the cause of death in 68% of patients after out-of-hospital cardiac arrest and in 23% of patients after in-hospital cardiac arrest [1]. More than half of the survivors have varying degrees of permanent brain injuries [2]. Despite recent advancements in the field of cardiac arrest and resuscitation, the management and prognosis of post-cardiac arrest brain injuries remain suboptimal and require further investigation [3].

Brain-derived neurotrophic factor (BDNF), a member of the neurotrophin family, is widely expressed in the central nervous system (CNS). BDNF binds to neurotrophin (NT) receptors and activates several neuroprotective pathways [4]. There are two types of NT receptors: the original high-affinity myosin receptor kinase (tropomyosin receptor kinase, Trk) and low-affinity receptor of p75 (p75 NT receptor, p75NTR). Both receptors are involved in the regulation of growth, differentiation, repair, apoptosis and survival of cells [5]. Several studies show that the signal transductions involved in Trk and p75NTR have mutually-antagonistic relationship [6]. Trk receptors generally mediate “positive” signals, such as promoting the growth of neurons to ensure their survival [7]. As a BDNF-specific receptor of the Trk family, TrkB belongs to tyrosine protein kinases with extracellular, transmembrane, and intracellular regions. Because the receptor tyrosine kinase domain is in its intracellular region, ligand and receptor binding can induce receptor dimerization and activation of the tyrosine kinase [8]. p75NTR has various biological effects. It promotes neuronal survival and growth, induces neuronal apoptosis [9], inhibits the axonal growth of neurons [10], and regulates cell cycle [11]. Although p75NTR mediates both “positive” and “negative” effects, it mainly mediates “negative” pro-apoptotic effects [12].

Endogenous hydrogen sulfide (H_2_S), a signaling gas molecule, is involved in ischemia/reperfusion injury [13] and shock development [14]. However, H_2_S can also protect nerve cells [15]. H_2_S increases intracellular Ca^2+^ and induces Ca^2+^ waves in primary cultures of astrocytes as well as hippocampal slices. H_2_S modifies hippocampal long-term potentiation (LTP) and functions as a neuromodulator. Studies show that H_2_S protects PC12 cells (neurocytes derived from pheochromocytoma of adrenal medulla of rat) [16] and hippocampal slices [17] against oxidative damage and toxicity by regulating BDNF-TrkB pathway, suggesting a close association of H_2_S and BDNF-TrkB pathway in CNS. Here we hypothesized that H_2_S may have impact on rats’ survival after CA and ROSC by regulating BDNF/TrkB/p75NTR pathway.

In our study, we established CA rat model using transcutaneous electrical stimulation. We modulated the endogenous H_2_S level in CA rats after CPR by intravenous injection of NaHS, and measured the expressions of BDNF, TrkB and p75NTR in brain tissues to investigate cause-and-effect relationship between NaHS, BDNF, TrkB and rats’ survival.

## Materials and methods

### Experimental groups

**Fig. 1:**
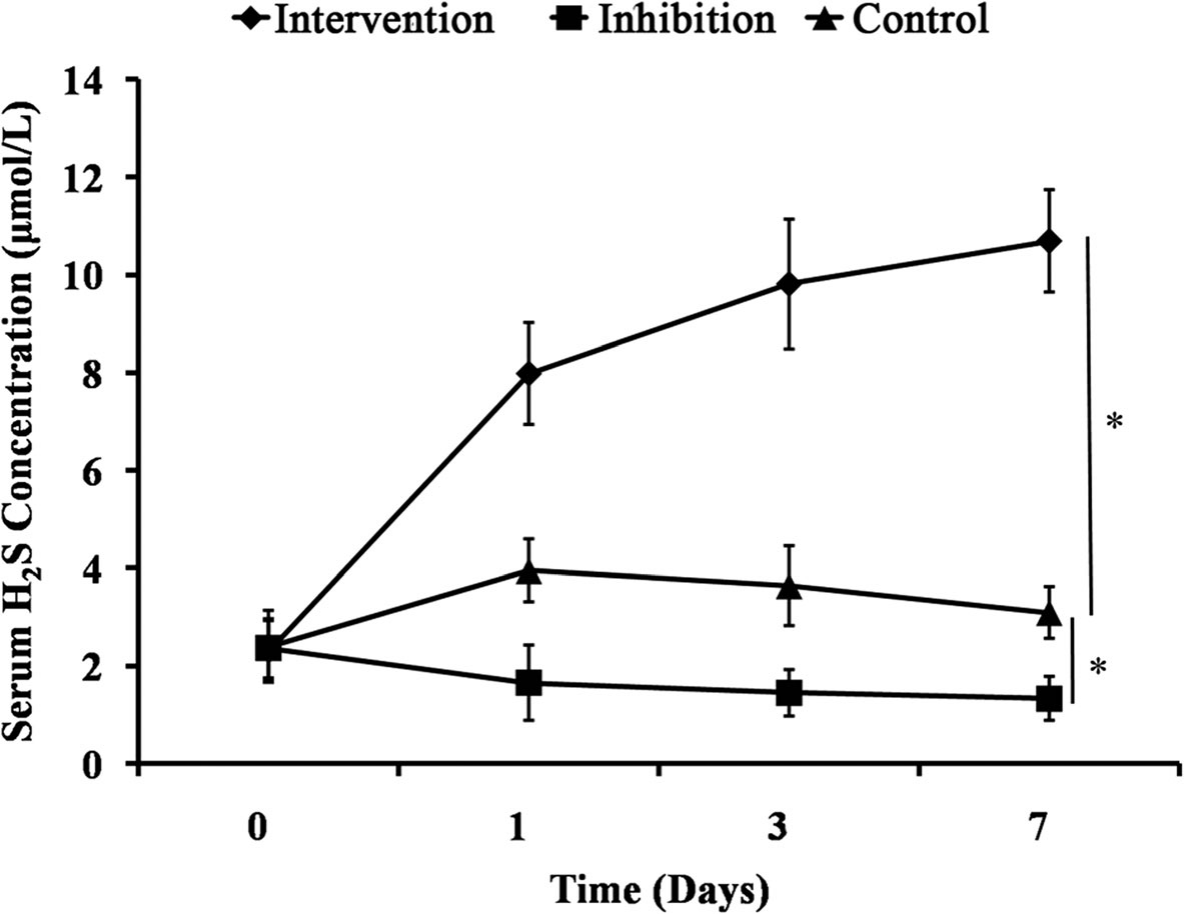
Serum hydrogen sulfide (H_2_S) concentrations in rats after cardiac arrest and cardiopulmonary resuscitation following restoration of spontaneous circulation. *p < 0.05

All animal experiments were performed according to the American experimental animal use guidelines (NIH Publications No. 80–23) and approved by the animal ethics committee of Xiamen University. Healthy male Sprague-Dawley rats (*n* = 240; age of 6–12 months; weight of 300–500 g) were provided by the experimental animal center of Xiamen University. The rats were divided into three groups: Intervention (*n* = 80), Inhibition (*n* = 80) and Control (*n* = 80). Twelve CA/CPR rats with successful ROSC were randomly and double-blindly selected from the surviving rats of these three groups on days 0, 1, 3, and 7, and served as subgroups for the result analysis. If there were more than 12 rats on the 7th day, only 12 rats were included in the 7-day subgroups. The general physiological characteristics of rats are shown in Table 1 and the rats’ survivals data are shown in Fig. 1.

**Table 1:** The baseline characteristics of the rats in all of the subgroups after restoration of spontaneous circulation (x ± s)

					Temperature (°C)	Respiratory rate (breaths/min)	Heart Rate (beats/min)	Systolic blood pressure (mmHg)	Diastolic blood pressure (mmHg)	Mean arterial pressure (mmHg)
	Subgroup	Amount	Age (M)	Weight (g)						
Intervation group	0 d	12	8.9 ± 1.7	414.9 ± 46.8	38.1 ± 0.6	74.9 ± 5.6	402.1 ± 32.4	113.7 ± 17.8	82.8 ± 15.7	108.2 ± 13.6
	1 d	12	8.8 ± 1.9	416.1 ± 42.8	37.9 ± 0.4	73.5 ± 5.9	412.2 ± 35.1	114.6 ± 19.3	81.7 ± 18.1	109.4 ± 17.3
	3 d	12	9.1 ± 1.6	408.1 ± 48.4	38.5 ± 0.5	72.7 ± 3.8	421.3 ± 37.8	114.1 ± 13.4	80.9 ± 13.5	111.0 ± 18.6
	7 d	12	9.2 ± 1.3	413.3 ± 45.7	38.2 ± 0.7	74.4 ± 6.1	417.3 ± 29.6	115.2 ± 24.8	84.1 ± 19.2	108.9 ± 12.6
Inhibition group	0 d	12	8.9 ± 1.6	416.6 ± 52.4	37.8 ± 0.2	73.8 ± 3.7	399.4 ± 29.4	112.8 ± 21.6	81.9 ± 16.8	108.4 ± 113
	1 d	12	9.4 ± 1.5	413.9 ± 49.2	37.6 ± 0.3	73.8 ± 4.1	412.1 ± 34.3	114.6 ± 17.8	82.6 ± 17.2	110.5 ± 12.6
	3 d	12	9.0 ± 1.4	409.9 ± 44.5	38.2 ± 0.9	75.6 ± 5.2	406.2 ± 25.7	115.3 ± 17.5	82.9 ± 14.2	109.1 ± 16.3
	7 d	12	9.0 ± 2.0	421.9 ± 49.2	38.3 ± 0.4	74.8 ± 4.2	409.3 ± 25.7	113.7 ± 18.3	83.1 ± 14.6	106.0 ± 12.2
Control group	0 d	12	8.3 ± 1.6	415.3 ± 47.4	37.8 ± 0.6	75.4 ± 4.4	417.8 ± 39.2	114.0 ± 28.4	86.1 ± 18.0	112.4 ± 18.4
	1 d	12	9.7 ± 1.6	412.9 ± 45.1	37.7 ± 0.7	74.6 ± 5.2	412.5 ± 38.6	115.1 ± 35.5	84.7 ± 16.0	109.2 ± 12.6
	3 d	12	7.8 ± 1.8	410.6 ± 43.7	38.4 ± 0.4	73.8 ± 3.6	406.4 ± 32.3	115.3 ± 34.6	85.1 ± 16.6	111.6 ± 19.4
	7 d	12	8.8 ± 1.8	421.3 ± 51.4	38.0 ± 0.2	74.9 ± 5.4	398.7 ± 21.7	114.5 ± 11.8	86.7 ± 14.2	108.0 ± 15.3
*P*			0.416	0.236	0.165	0.356	0.079	0.061	0.126	0.213

### Establishment of CA/CPR rat model, ROSC and samples preparation

CA rat model was established based on our previously reported method [18]. The ROSC rats received intervention immediately after ROSC as described in Section 2.3. ECG and hemodynamic monitoring were performed for 4 h. During the 4 h monitoring period, the rats with weak spontaneous breathing (PaCO2 < 60 mm mmHg) were continuously mechanically ventilated without any treatment. The breathing pattern was assessed every 15 min to decide whether to continue the mechanical ventilation [18]. If the rats were awake during the procedure, we intraperitoneally injected 3% sodium pentobarbital (30 mg/kg). After 4 h, mechanical ventilation was stopped, all tubes were removed and wounds were sutured. The rats were observed for 7 d. All the selected rats were intraperitoneally injected with 3% sodium pentobarbital (30 mg/kg). Cardiac puncture method was used to obtain 2–5 mL blood. All of the rats were killed by decapitation, and 3 mm brain tissues including cortex, hippocampus, and cerebellum were harvested, fixed in formaldehyde, and embedded in Paraffin.

### Intervention methods

The rats in Intervention group were intravenously injected with 1 mL sterile NaHS (a donor for H2S, CAS: 140650–84-6, Sigma, USA) at a dose of 14 μmoL/kg·d at 1 h after ROSC. The rats in Inhibition group were intravenously injected with 1 mL sterile diluted hydroxylamine (an inhibitor for H2S, CAS 7803-49-8, Sigma) at a dose of 40 μmoL/kg·d at 1 h after ROSC. The doses of NaHS and hydroxylamine were chosen based on our previous study and other references [19] [20] [21]. The rats in Control group received 1 mL sterile saline at 1 h after ROSC.

### Specimen collection

Before all the rats were euthanized, 1 mL of femoral vein blood sample was collected and added into 1 mL Eppendorf (EP) tubes with a coagulant agent. The blood samples were centrifuged at 4000 r/min for 15 min at 4 °C. The supernatant was collected, coded, and stored at − 80 °C for serum H_2_S determination.

For each subgroup, six rats were perfused with saline through the heart, followed by 4% paraformaldehyde for 30 min. The brains were removed, fixed in 4% paraformaldehyde, embedded in paraffin, and sectioned for immunohistochemistry assay. For the other six rats, hippocampus tissues were harvested, snap-freezing in liquid nitrogen, and stored at − 80 °C for quantitative real-time PCR and Western blot analyses.

### Serum hydrogen sulfide determination

A H_2_S kit (BC2055, Solarbio, Beijing China) was used to detect the H_2_S level. The following reagents were added in a 5 mL glass tube: 0.5 mL of 1% (*W*/W) zinc acetate, 2.5 mL of distilled water, 0.1 mL of serum sample or NaHS·H_2_O (CAS: 140650–84-6, Sigma), 0.5 mL of 20 mM (7.2 M HCl) dimethyl-*p*-phenylenediamine sulfate, and 0.4 mL of 30 mM (1.2 M HCl) ferric chloride. The reagents were mixed and transferred to an incubator at 37 °C for 20 min to allow a complete color change. The mixtures were added with 1 mL 10% trichloroacetic acid, and then diluted with distilled water for a final volume of 5 mL. The mixtures (5 mL) were centrifuged at 12,000 rpm for 10 min. The supernatants were collected and measured at an absorbance of 620 nm. Linear regression equation was used to calculate the serum H_2_S concentration. Each serum sample was measured 3 times, and the concentration of each sample was the mean of the three measurements. The final concentration (μmol/L) of H_2_S in each group was averaged and presented as mean ± standard deviation.

### Quantitative real-time PCR

The mRNA expressions of BDNF, TrkB, and p75NTR were measured by quantitative real-time PCR. Total RNA was isolated from the brain tissues using an RNeasy kit with Trizol (Invitrogen, USA), and reverse-transcribed into cDNA using a reverse transcription kit with M-MLV polymerase (Promega, USA). The sequence-specific primers used were as follows: BDNF-F, 5′-CGATTAGGTGGCTTCATAGGAG-3′ and BDNF-R, 5′-ACGAACAGAAACAGAGGAGAGATT-3′; TrkB-F, 5′-CAAGTTGGCGAGACATTCCA-3′ and TrkB-R, 5′-AGTCATCGTCGTTGCTGATGAC-3′; p75NTR-F, 5′-TTCCTTAGCCCCTCCCTTCT-3′ and p75NTR-R, 5′-CCTGCCTTTCTCTGGGTTTTAC-3′; ACTIN-F, 5′-GCTATGTTGCCCTAGACTTCGA-3′ and ACTIN-R, 5′-GATGCCACAGGATTCCATACC-3′. PCR was performed using an IQ5 PCR System (Bio-Rad, USA) with an initial denaturing step at 95 °C for 15 s, 45 cycles of denaturing at 95 °C for 5 s, and annealing at 60 °C for 30 s. The relative expressions of genes were determined using the ΔΔCt method [22] to normalize the target gene expression to that of the housekeeping gene (ACTIN).

### Western blot

The protein expressions of BDNF, TrkB, and p75NTR were measured by Western blot. Lysis buffer [400 μL; 50 mM Tris-HCl, pH 7.4, 150 mM NaCl, 1% nonidet P-40, 0.5% deoxycholic acid, 0.1% sodium dodecyl sulfate (SDS), 5 mM ethylenediaminetetraacetic acid, 2 mM phenylmethylsulfonyl fluoride, 20 μg/mL of aprotinin, 20 μg/mL of leupeptin, 10 μg/mL of pepstanin A, and 150 mM benzamidine] was added into 100 mg of brain tissues, and homogenized for 30 min on ice. The homogenate was transferred into 1.5 mL EP tubes and centrifuged at 12,000 rpm for 5 min at 4 °C. The supernatant was collected. Protein quantification was performed using the bicinchoninic acid method, and SDS-polyacrylamide gel electrophoresis was performed. The proteins were transferred onto polyvinylidene fluoride membranes, probed with appropriate primary and secondary antibodies, and then detected using a Pierce Fast Western Blot Kit ECL Substrate (Cat No. 35055, Thermo, USA). The primary antibodies (1:1000 dilution) used were as follows: rabbit anti-rat BDNF (H-117) (sc-20,981, Santa Cruz Biotechnologies, USA), rabbit anti-rat TrkB (H-181) (sc-8316, Santa Cruz Biotechnologies), rabbit anti-rat p75NTR (H-92) (sc-5634, Santa Cruz Biotechnologies), and rabbit anti-rat α-tubulin (sc-5546, Santa Cruz Biotechnologies). The secondary antibody (1:5000) used was goat anti-rabbit IgG (sc-2004; Santa Cruz Biotechnologies). Immunoreactivity was imaged using Perfection 3490 photo gel imaging systems (Epson, Japan) and analyzed using Image Pro PLUS (Media Cybernetics, USA). Protein expression was normalized to tubulin.

### Immunohistochemistry

The protein expressions of BDNF, TrkB and p75NTR in the cortex, hippocampus, and cerebellum were detected by immunohistochemistry. Paraffin-embedded tissues were sectioned at a thickness of 10 μm and mounted onto a 1.35-μm thin polyethylene film (PALM GmbH; Wolfratshausen, Germany) overlaid on a glass slide. Sections were incubated with primary antibodies overnight at 4 °C. After wash, the sections were incubated with biotinylated goat anti-rabbit IgG (1:5000, sc-2004; Santa Cruz Biotechnologies) for 30 min. Positive staining was revealed using diaminobenzidine [SABC immunohistochemical staining kit (Cat. No. SA1025), Wuhan Boster Biological Engineering Co., Ltd., China)] according to the manufacturer’s instructions. The primary antibodies (1:1000 dilution) used were as follows: rabbit anti-rat BDNF (H-117) (sc-20,981, Santa Cruz Biotechnologies), rabbit anti-rat TrkB (H-181) (sc-8316, Santa Cruz Biotechnologies), and rabbit anti-rat p75NTR (H-92) (sc-5634, Santa Cruz Biotechnologies). The immunostained slides were imaged using Imaging-Pro Plus 6.0 [23]. Five fields were randomly selected in each section for integrated optical density (IOD) calculations. The IOD of each slice was the mean IOD of the five fields.

### Statistical analysis

SPSS 13.0 statistical package was used for statistical analysis. All the values were presented as mean ± standard error. Multi-comparisons between means were analyzed by the Student-Newman-Keuls method. *p* < 0.05 was considered statistically significant. Survival data were analyzed by Kaplan-Meyer Analysis method and Log Rank (Mantel-Cox) was used to compare the survival difference.

## Results

### General physiological characteristics of rats

A total of 240 rats were used for the CA/CPR model. However, some rats died during ROSC or within the 7-d observation period. Thus, only 12 rats were selected on days 0, 1, 3, and 7 for the final analysis. The general physiological characteristics of the rats are shown in Table 1. After ROSC, no significant difference was found among the baseline characteristics of the rats in all the subgroups (*p* > 0.05).

### Comparison of serum H_2_S concentrations

The average serum H_2_S concentrations of all the subgroups were calculated (Fig. 1). There was no significant difference in the average H_2_S concentrations on day 0 (*p* > 0.05). After NaHS·H_2_O stimulation, the serum H_2_S concentrations in the Intervention group gradually increased over time (Fig. 1) and were significantly higher than the Control group (*p* < 0.05). Because the H_2_S formation in the Inhibition group was inhibited by hydroxylamine (Fig. 1), the serum H_2_S concentrations in Inhibition group was significantly lower than the Control group (*p* < 0.05), and were maintained at a low level during the 7-d observation period.

### Kaplan-Meyer analysis of survival rate

**Fig. 2:**
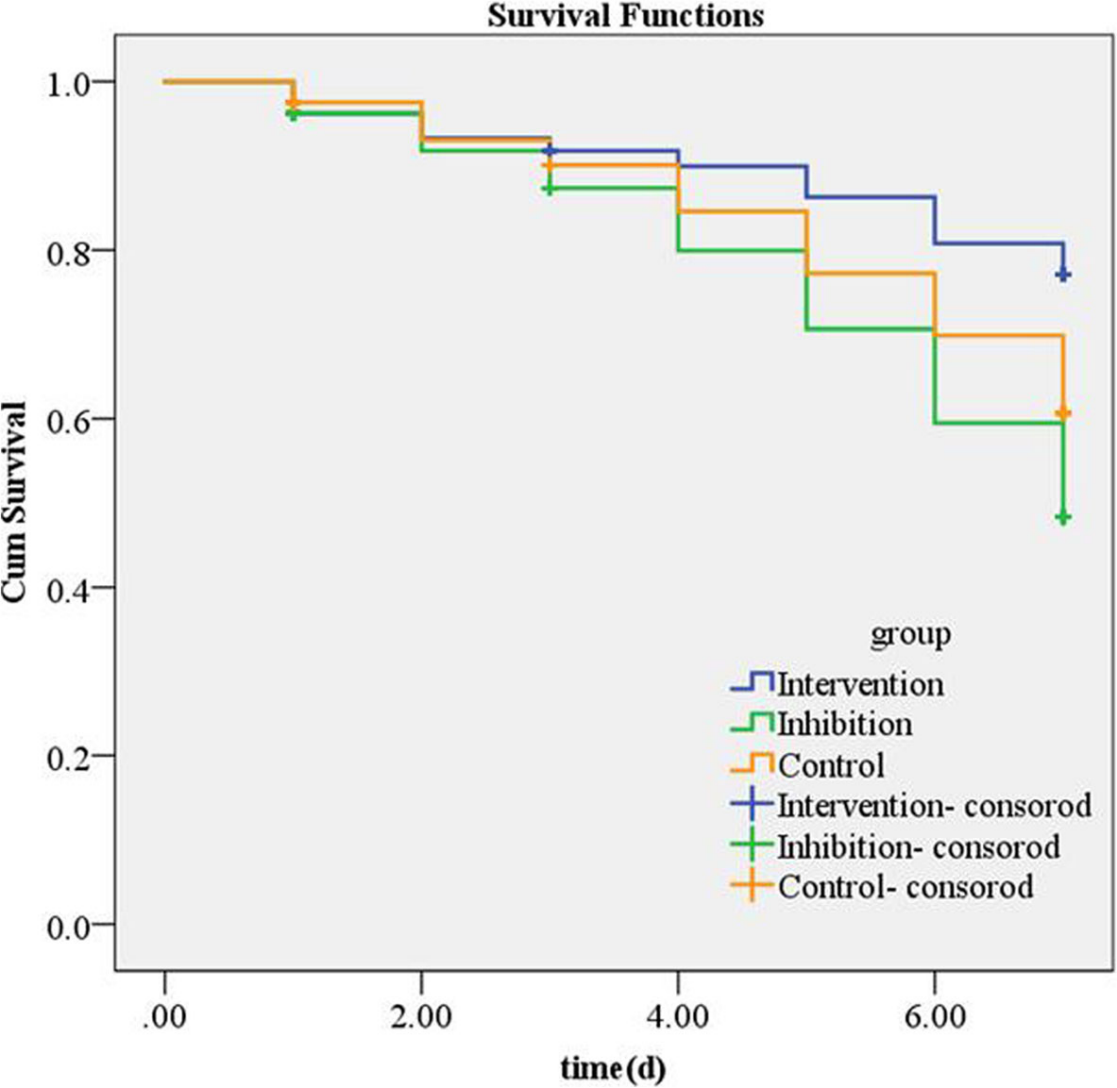
Kaplan-Meyer Analysis of survival rate. The survival curve indicates that the Intervention group (blue line) had the highest survival rate, compared to the Inhibition group (green line) and the Control group (yellow line). The analysis was performed from day 0 to day 7 (censored)

Survival rate was 82.5% (66/80) in Intervention group, 62.5% (50/80) in Inhibition group, and 71.3% (57/80) in Control group. The 95% CI of survival time was 6.015~6.753 days in Intervention group, 5.429~6.281 days in Inhibition group, and 5.728~6.520 days in Control group, which had a significant difference (χ^2^ = 8.683, *P* = 0.013) (Fig. 2).

### Comparison of the relative mRNA expression levels of BDNF, TrkB, and p75NTR in the brain tissues

**Fig. 3:**
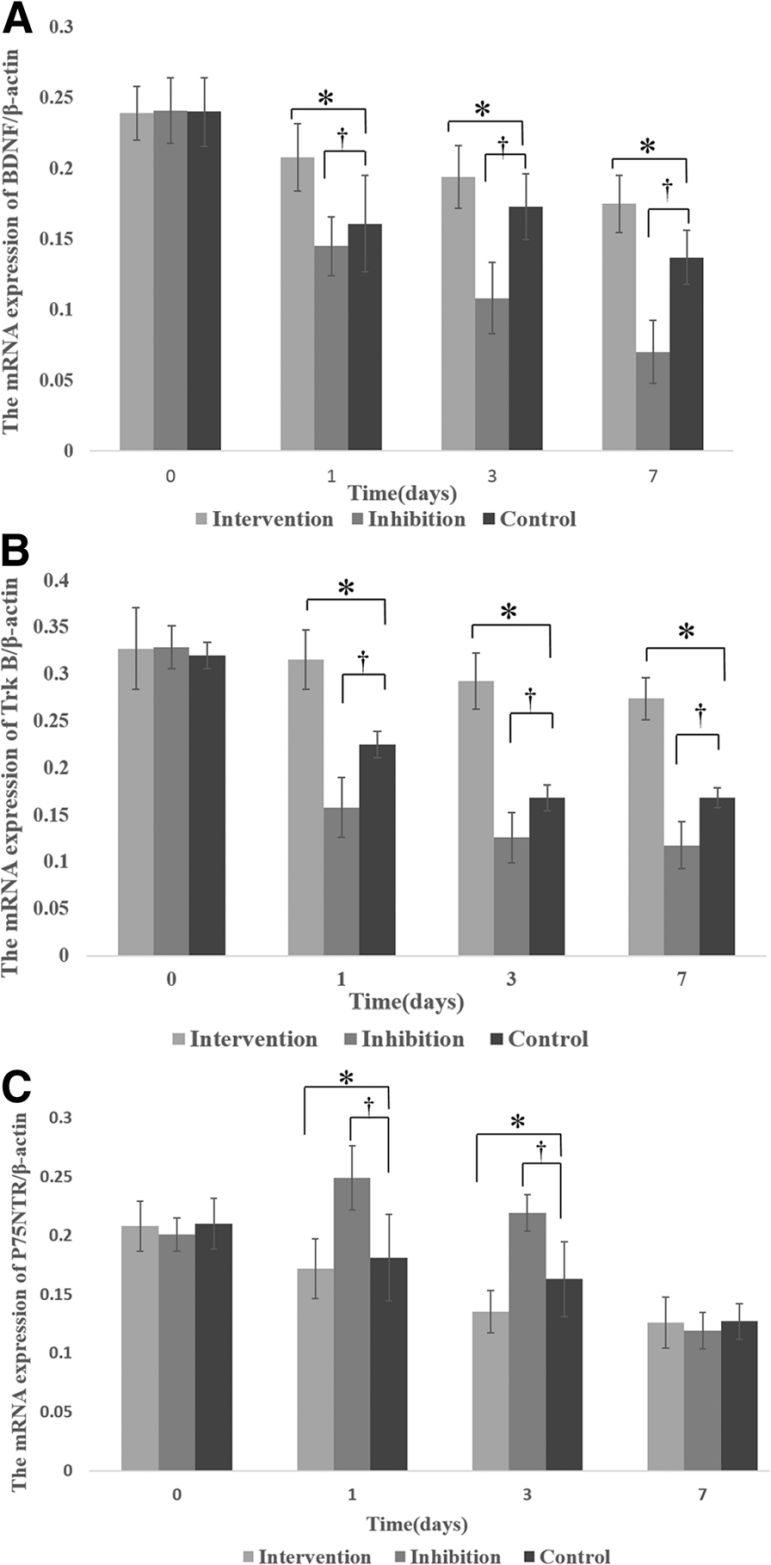
Comparison of the relative mRNA expression of (**a**) BDNF, (**b**) TrkB, and (**c**) p75NTR in brain tissues. *p < 0.05, Intervention versus Control; ^†^*P* < 0.05, Inhibition versus Control

The relative mRNA expression levels of BDNF, TrkB and p75NTR in the brain tissues of CA/CPR rats after ROSC decreased over time during the 7-d observation period (Fig. 3). There was no significant difference in mRNA expression of BDNF, TrkB, and p75NTR among the three groups at 0-d after CPR (Fig. 3a-c). The mRNA levels of BDNF and TrkB were similar on days 0, 1, 3, and 7 in the Intervention group (Fig. 3a, b) (*p* > 0.05). However, the mRNA levels of BDNF and TrkB on days 1, 3, and 7 in the Intervention group were significantly higher than the Control group (*p* < 0.05). In contrast, the mRNA levels of BDNF and TrkB decreased over time in the Inhibition group and were significantly lower than the Control group (*p* < 0.05). The mRNA levels of p75NTR in the Inhibition group were significantly higher on days 1 and 3 than day 0, and also significantly higher than the Control group on days 1 and 3. In contrast, the mRNA level of p75NTR in the Intervention group decreased daily, and significantly decreased on days 1 and 3 compared with the Control group (*p* < 0.05).

### Relative protein expressions of BDNF, TrkB, and p75NTR in brain tissues

**Fig. 4:**
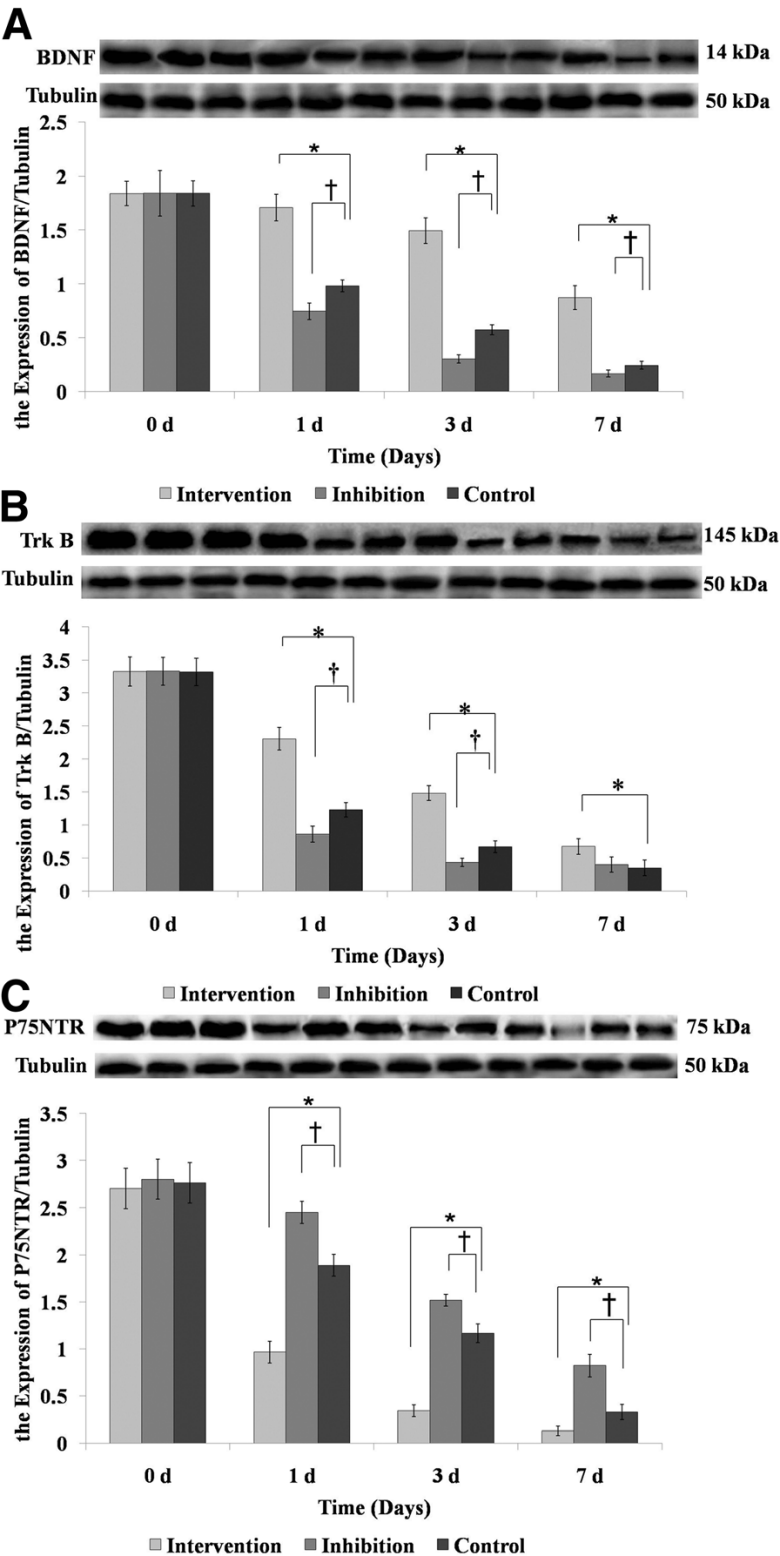
Comparison of the relative protein expression of (**a**) BDNF, (**b**) TrkB, and (**c**) p75NTR in brain tissues. *p < 0.05, Intervention versus Control; ^†^*P* < 0.05, Inhibition versus Control

There was no significant difference in the relative protein expressions of BDNF, TrkB, and p75NTR among the three groups on day 0 (Fig. 4). The expressions of BDNF and TrkB in the Intervention group decreased over time (Fig. 4a and b), but were significantly higher than the Control group on days 1, 3, and 7 (*p <* 0.05). The relative protein expressions of BDNF and TrkB in the Inhibition group decreased significantly than the Control group (*p* < 0.05) over time. In addition, the relative protein expression of p75NTR in the Intervention group significantly increased on days 1, 3, and 7, compared with the Control group (*P* < 0.05). In contrast, the relative protein expression of p75NTR in the Inhibition group reduced significantly compared with the Control group (*p* < 0.05).

### Immunohistochemical staining of BDNF, TrkB, and p75NTR in the cerebral cortex, hippocampal tissue, and cerebellum

**Fig. 5:**
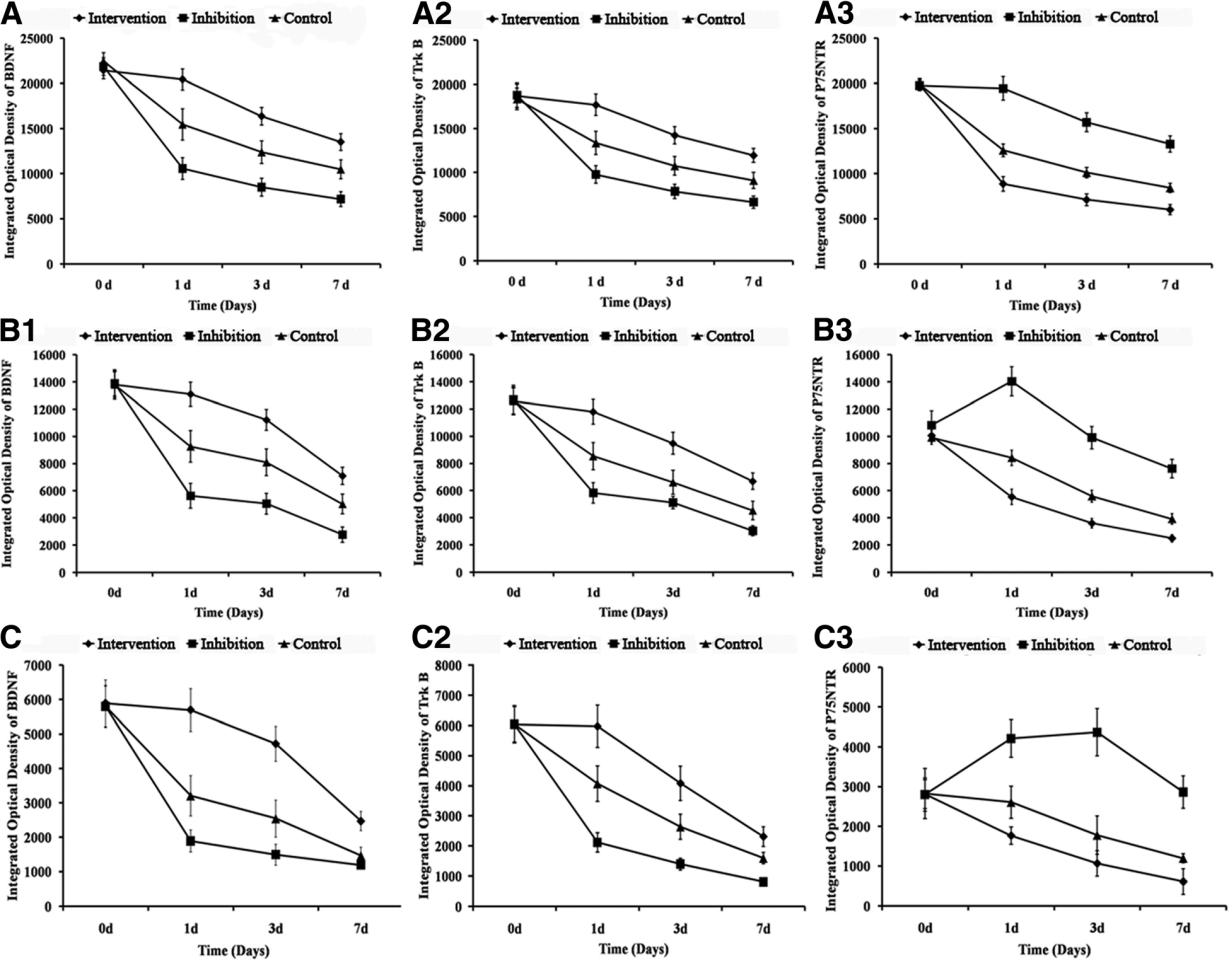
Integrated optical density of BDNF, TrkB, and p75NTR in cerebral cortex (A1, A2, and A3), hippocampal tissues (B1, B2, and B3) and cerebellum (C1, C2, and C3)

**Fig. 6:**
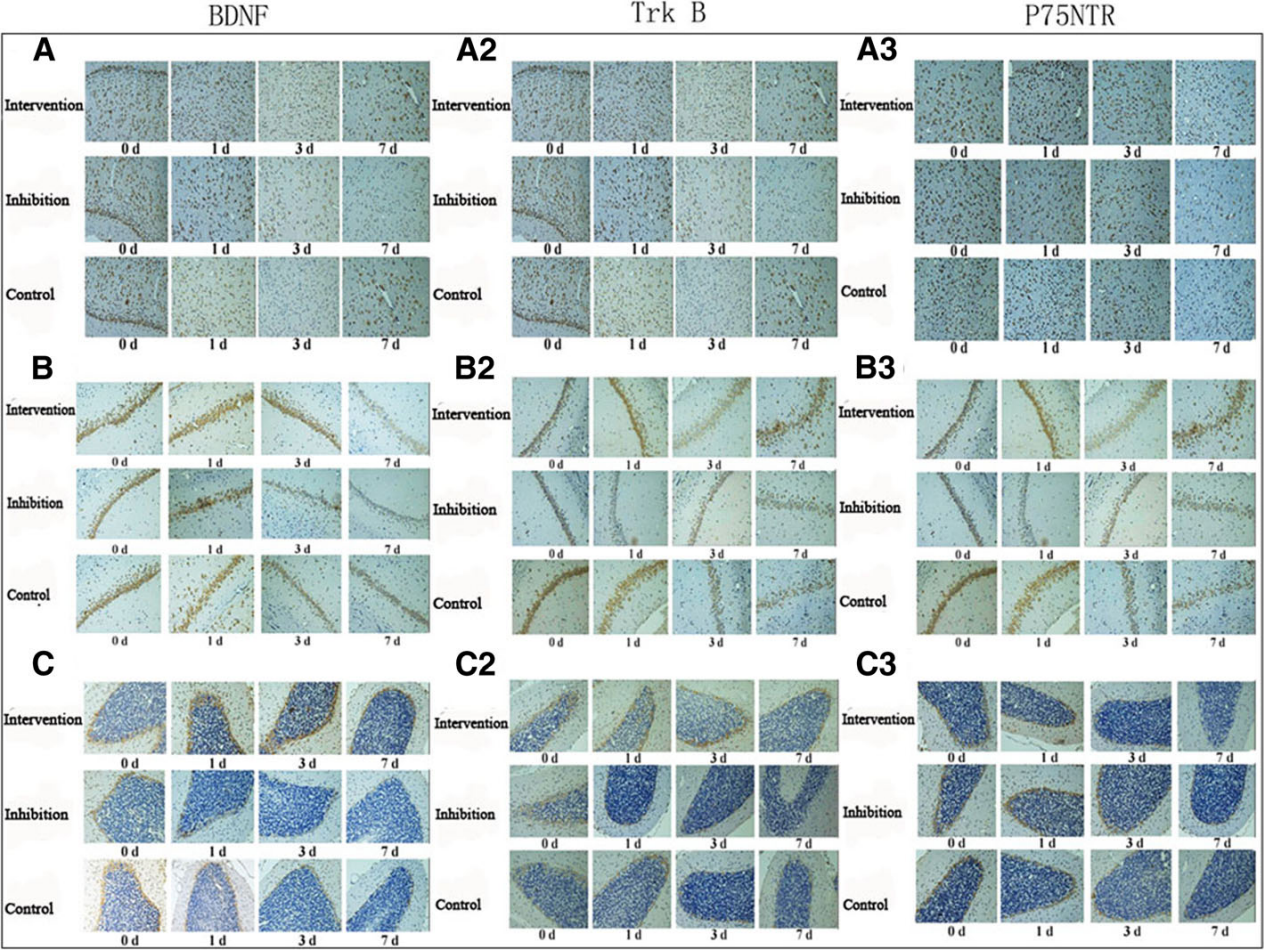
Immunohistochemical staining of BDNF, TrkB, and p75NTR in cerebral cortex (A1, A2, and A3), hippocampal tissues (B1, B2, and B3) and cerebellum (C1, C2, and C3)

The expressions of BDNF, TrkB and p75NTR in the cerebral cortex, hippocampal tissue, and cerebellum of CA/CPR rats after ROSC decreased over time during the 7-d observation period (Figs. 5 and 6). There was no significant difference in the IODs of BDNF, TrkB, and p75NTR in the cerebral cortex (Fig. 5A1-A3 and Fig. 6 A1-A3), hippocampal tissue (Fig. 5B1-B3 and Fig. 6B1-B3), and cerebellum (Fig. 5C1-C3 and Fig. 6C1-C3) among the three groups at 0-d after CPR. These findings are consistent with the imaging results (Fig. 6). On days 1, 3, and 7, the IODs of BDNF and TrkB significantly increased in the Intervention group (*p* < 0.05), but significantly decreased in the Inhibition group (*p* < 0.05), compared with the Control group. In contrast, the IOD of p75NTR significantly decreased in the Intervention group (*p* < 0.05), but significantly increased in the Inhibition group (*p* < 0.05) on days 1, 3, and 7.

## Discussion

A post-cardiac arrest syndrome due to ischemic encephalopathy occurs after cardiac arrest, which aggravates sickness and often results in death [24]. Most CA patients after ROSC become comatose or suffer from chronic neurological disability [25]. A high percentage of survivors exhibit permanent cognitive dysfunction and poor social behavior with a lower quality of life [26]. During CPR, brain damage can occur during ischemia and reperfusion periods due to the interrupted cerebral blood flow. Although the mechanisms involved in ischemia and reperfusion periods differ, nerve cell death occurs during both of these two periods [27]. Early intervention, which is used to delay the onset of cell death, can increase the survival rate of patients.

H_2_S can protect nerve cells by improving the blood circulation in the brain [28]. Studies show that physiological H_2_S concentration can clear peroxynitrite (ONOO–), reduce neuronal damage mediated by ONOO– [29], and inhibit oxidative stress to protect the nervous system by increasing mitochondrial reduced glutathione (GSH) levels [30]. In addition, intravenous injection of NaHS [H_2_S donor [31]] can prevent neuronal damage [32] and hypochlorous acid-induced PC12 cell injury [33]. Our results indicate that intravenous injection of NaHS·H_2_O effectively increased the H_2_S concentration in the serum of CA/CPR rats after ROSC; while hydroxylamine inhibited H_2_S formation. These findings are consistent with those of Wallace et al. [34]. Meanwhile, Kaplan-Meyer analysis showed that the survival time of the Intervention group was significantly higher than the Control group, while the survival time of the Inhibition group was significantly lower than the Control group. All the above results indicate that NaHS·H_2_O injection can improve the survival time after ROSC, while hydroxylamine can accelerate the death.

As a member of the neurotrophic factor family, BDNF is widely distributed in the nervous system, the endocrine system, and other organs and tissues. BDNF is mainly expressed in the neurons of the nervous system, with the highest concentrations found in the cerebral cortex and hippocampus. BDNF plays an important role in neuronal survival, differentiation, growth, and development. BDNF also plays a crucial role in preventing neuronal injury, improving the pathological state of neurons, and promoting the regeneration of injured neurons. Our results demonstrate that intravenous injection of NaHS·H_2_O effectively increased BDNF expression, while hydroxylamine inhibited BDNF expression. H_2_S is considered as an antioxidant that can decrease oxidative stress [35] and increase BDNF expression in the hippocampus of rat model of brain injury [17].

Recent studies have shown that the expressions of BDNF and its receptor TrkB increase in ischemia/reperfusion injury of the brain [36, 37]. BDNF can inhibit caspase-3 activity and regulate the expressions of bcl-2 and bax to inhibit cell apoptosis [38]. TrkB upregulation enhances the effect of BDNF on neuronal growth. Moreover, the activation of TrkB receptor can block the effects of intracellular damage factor on protein kinase C activity to prevent neuronal degeneration and necrosis [39]. p75NTR expression also increases during injury, and inhibiting p75NTR signaling can delay disease progression and promote cell survival [40]. p75NTR-induced apoptosis is obtained by binding with BNDF and activating the c-Jun and N-terminal kinase pathway [41]. In this study we investigated the impact of NaHS-induced changes in BDNF and its receptor (TrkB and p75NTR) on rats’ survival after CA and ROSC. Our results suggest that intravenous injection of NaHS·H_2_O can effectively increase TrkB expression and inhibit p75NTR expression; whereas, hydroxylamine had the opposite effects on TrkB and p75NTR expressions.

In conclusion, in this study we demonstrate that intravenous injection of NaHS·H_2_O can increase the level of BDNF and TrkB, while decrease p75NTR in the brain tissues of CA/CPR rats after ROSC, leading to an increased survival time of the rats. In contrast, hydroxylamine (cystathionine-β-synthase inhibitor) decreased BDNF and TrkB levels and increased p75NTR, leading to decreased survival time of the rats. Therefore, NaHS may have neuroprotective effects by promoting the BDNF/TrkB signaling pathway.

### Limitations

There are some limitations in this study. Firstly, the age of the rats was variable (6–12 months). However, we did not find an association between the age and the survival time of the CA model. Secondly, we only demonstrate that H_2_S can modulate the levels of BDNF and its receptors in ROSC rats, leading to an increased survival time; however, the underlying mechanisms need to be investigated in our future studies.
